# Understanding Type 2 Diabetes Mellitus and the Knowledge Gaps among Future Health Care Professionals: Insights from Saudi Arabia

**DOI:** 10.1055/s-0045-1812884

**Published:** 2025-11-13

**Authors:** Zeeshan Feroz, Ismail Memon, Nader Alharbi, Abdulmohsen Alkushi, Yazeed Alrayani

**Affiliations:** 1 Department of Basic Sciences, College of Science and Health Professions, King Saud bin Abdulaziz University for Health Sciences, Riyadh, Kingdom of Saudi Arabia; 2 King Abdullah International Medical Research Center, King Saud bin Abdulaziz University for Health Sciences, Ministry of National Guard-Health Affairs, Riyadh, Saudi Arabia; 3 Department of Human Body Structure and Function, Saba University School of Medicine, Church Street, Saba, Netherlands; 4 Department of Basic Medical Sciences, College of Medicine, King Saud bin Abdulaziz University for Health Sciences, Riyadh, Saudi Arabia

**Keywords:** awareness, diabetes mellitus, knowledge, undergraduate students

## Abstract

**Background:**

Saudi Arabia ranks seventh in the world for diabetes prevalence, yet many individuals are unaware of the disease. Increasing awareness, particularly among future health professionals who are often consulted for guidance, is essential to reduce the burden of diabetes. It is needed; to what extent do preprofessional health sciences students in Saudi Arabia understand type 2 diabetes mellitus (T2DM), including prevention, screening, and treatment? This study aimed to assess the knowledge and awareness of T2DM among preprofessional health sciences students in Saudi Arabia, with a focus on prevention, screening, and treatment.

**Methods:**

A descriptive cross-sectional study was conducted among 429 health professions students (required sample size: 317). The questionnaire included demographic data followed by 47 questions assessing knowledge of diabetes. Descriptive statistics were used to summarize demographic characteristics, while logistic regression with the forward-backward with early dropping variable selection algorithm (R package MXM) was applied to analyze the influence of demographic factors on knowledge scores.

**Results:**

Out of the 429 participants, 63.6% were male and 36.4% were females. The majority (92.1%) were aged 20 years or younger, with 60.1% being first-year students. A notable percentage (78.1%) reported having diabetic relatives. Most participants (96.3%) recognized that diabetes is preventable; however, 86.7% mistakenly thought that prediabetes is irreversible. While over 90% exhibited good understanding of the symptoms and risk factors of diabetes, 63.6% were unaware of the role of oral hypoglycemic agents in treatment. Conversely, knowledge regarding the use of insulin e was well comprehended by 93.0% of the respondents.

**Conclusion:**

The study suggests that health professional students in Saudi Arabia have a good understanding of type 2 diabetes; however, they exhibit a deficiency in knowledge about the use of oral hypoglycemic agents and the potential for reversing prediabetes. By addressing these gaps through curricular enhancements, future health care professionals could be better prepared to confront the escalating diabetes epidemic in the region.

## Introduction

Chronic noncommunicable diseases (NCDs) represent a significant threat to global public health. They are the leading cause of mortality worldwide, responsible for approximately 41 million deaths annually, which constitutes 71% of all deaths. Among the various NCDs, diabetes mellitus (DM) ranks fourth, resulting in approximately 1.6 million deaths globally each year. 1

DM, a prominent category of NCDs, is experiencing a troubling increase in prevalence. Current estimates suggest that around 536.6 million individuals (10.5% of the global population) are living with diabetes, a figure projected to rise to 783.2 million (12.2%) by 2045. 2 According to the World Health Organization, Saudi Arabia has the second-highest prevalence of diabetes in the Middle East and ranks seventh globally. Approximately 8.5% of the Saudi population is estimated to be living with diabetes, with prevalence rates continuing to rise in recent years. 3 Type 2 DM (T2DM) affects individuals across all age groups; however, it is increasingly prevalent among adolescents and young adults (ages 15–34). 4 Fortunately, T2DM is preventable if risk factors are identified early. 5 Enhancing awareness of these risk factors in the general population, particularly among health care professionals who are often consulted for guidance, can help reduce the burden of T2DM and its complications.

It is therefore essential that health sciences students, as future health care providers, are adequately informed about the risk factors, preventive strategies, and management of DM. The extent to which preprofessional health sciences students in Saudi Arabia understand T2DM, however, remains unclear.

This study aimed to evaluate the knowledge and awareness of T2DM including risk factors, treatment, and the reversibility of prediabetes among students at King Saud bin Abdulaziz University for Health Sciences (KSAU-HS) in Riyadh, Saudi Arabia. The focus was on their understanding of risk factors, clinical information, preventive measures, and perceptions of the disease.

## Methods

### Ethical Approval

This study was approved by the Research Committee and the Institutional Review Board (IRB) at King Abdullah Medical Research Center, Riyadh, Saudi Arabia (Reference No. IRBC/0320/20). Informed consent was obtained from all participants during data collection.

### Study Design and Participants

A descriptive cross-sectional survey design was employed using a self-administered structured questionnaire. The study population comprised first- and second-year preprofessional students (male and female) from the College of Science and Health Professions (COSHP) at KSAU-HS, Riyadh campus, during the 2021 to 2022 academic year.

All first- and second-year COSHP students were invited to participate. They received a briefing regarding the study's objectives and instructions for completing the questionnaire. Participation was voluntary and anonymous. Questionnaires were distributed along with a consent form outlining the purpose of the research, and participants were assured of confidentiality.

### Development of the Questionnaire

The structured questionnaire was developed following a comprehensive literature review, drawing on studies conducted by Khan et al, Xu et al, and Amankwah-Poku. 5 6 7 Validity of the questionnaire was established through face and content validity assessments. A panel of subject experts evaluated the questionnaire to ensure alignment with the study objectives.

A pilot study was conducted with 35 university students to identify potential issues with questions' design, flow, or interpretation. Based on feedback, minor modifications were made. The finalized version was then translated into Arabic by a bilingual individual with expertise in the subject area, and the translation was reviewed by a language expert prior to distribution.

The finalized questionnaire consisted of two sections. The first collected demographic data, including age, gender, year of study, and personal and familial health status. The second assessed knowledge of diabetes through 47 questions covering symptoms, risk factors, diagnosis, complications, treatment, and prevention.

### Sample Size

The required sample size was calculated to be 317, based on a 95% confidence interval, a 5% margin of error, and an estimated population proportion of 50%.

### Data Analysis

Descriptive statistics were used to summarize demographic characteristics expressed as frequencies and percentages or as means and standard deviations. Participants' responses to the knowledge questions were also summarized as percentages. To evaluate the influence of demographic variables on the participants' knowledge, the forward backward with early dropping variable selection algorithm 8 was implemented in the R package MXM. 9 Given that knowledge questions offered only two response options (Yes/No), logistic regression was employed. 10 The sign in parentheses accompanying each demographic variable indicates the direction of its effect.

The interrelationships among the 47 knowledge questions were further analyzed using a Bayesian network. 11 The PC Hill Climbing (PCHC) Bayesian network-learning algorithm, 12 as described in the literature, was employed for this analysis and implemented using the R package pchc. 13

## Results

A total of 429 students participated in the survey. The majority of the participants (92.10%) were aged 20 years or younger, with first-year students representing the largest group (60.1%). Additionally, a significant proportion of the respondents (78.1%) reported having family members or relatives with type 2 diabetes ( Table 1 ).

**Table 1:** Descriptive statistics of the demographic variables

Categorical variable	Values	Frequency (%)
Age	20 years or younger	395 (92.10)
	Older than 20 years	34 (7.90)
Gender	Female	156 (36.40)
	Male	273 (63.60)
Year of study	1st	258 (60.10)
	2nd	171 (39.90)
If you were a second-year student, then your group is:	PAMS	48 (28.07)
	PDNT	20 (11.70)
	PHIS	7 (4.09)
	PMED	78 (45.61)
	PPHR	18 (10.53)
Do you suffer from type 2 diabetes mellitus?	Don't know	21 (4.90)
	No	401 (93.50)
	Yes	7 (1.60)
Does any of your family member or relative has/had type 2 diabetes mellitus?	Don't know	17 (4.00)
	No	77 (17.90)
	Yes	335 (78.10)
Are you maintaining healthy diet?	Don't know	58 (13.50)
	No	280 (65.30)
	Yes	91 (21.20)
Are you engaged in any physical exercises?	Don't know	30 (7.00)
	No	248 (57.80)
	Yes	151 (35.20)

Abbreviations: PAMS, preapplied medical sciences; PDNT, predental; PHIS, prehealth informatics; PMED, premedicine; PPHR, prepharmacy.

The participants' responses to diabetes-related questions are summarized in Table 2 . Of the 47 questions, only 4 received a lower percentage of positive (“Yes”) responses. The majority of participants responded positively to a large number of questions ( *n* = 43).

**Table 2:** Distribution of responses to each diabetes-related question

No.	Full question	Answers	
		Yes %	No %
Q1	Diabetes is a medical condition characterized by elevated levels of glucose in the blood	71.10	28.90
Q2	Type 2 diabetes is preventable and controllable	96.27	3.73
**Q3**	Excessive eating is a symptom of diabetes	27.74	72.26
Q4	Excessive thirst is a common symptom observed in individuals with diabetes	91.14	8.86
Q5	Diabetes can be treated	76.79	23.21
Q6	Unusual weight loss occurs in diabetic patients	83.68	16.32
Q7	Persistent fatigue is a common symptom among individuals with diabetes	90.44	9.56
Q8	Frequent urination occurs in diabetic patients	97.20	2.80
Q9	Blurry vision occurs in diabetic patients	92.07	7.93
Q10	Slow healing of cuts and wounds occurs in diabetic patient	95.80	4.20
Q11	Numbness of hands and feet occurs in diabetic patient	92.31	7.69
Q12	Excessive Sugar intake increases the chances of developing diabetes	88.81	11.19
Q13	Sedentary life styles increase the chances of developing diabetes	92.07	7.93
Q14	Fatty food intake increases the chances of developing diabetes	85.08	14.92
Q15	Obesity increases the chances of developing diabetes	97.44	2.56
Q16	High blood pressure increases the chances of developing diabetes	81.12	18.88
Q17	Family history of diabetes increases the chances of developing diabetes	94.87	5.13
Q18	Being above 45 years old, increases the chances of developing diabetes	90.91	9.09
**Q19**	Race/ethnicity increases the chances of developing diabetes	29.37	70.63
Q20	Uncontrolled diabetes may cause eye problems (retinopathy)	92.31	7.69
Q21	Uncontrolled diabetes may cause cardiovascular diseases	91.61	8.39
Q22	Uncontrolled diabetes may cause nerve damage (neuropathy)	88.81	11.19
Q23	Uncontrolled diabetes may cause kidney damage (nephropathy)	89.51	10.49
Q24	Uncontrolled diabetes may cause diabetic foot (gangrene)	92.54	7.46
Q25	Uncontrolled diabetes may increase the chances of recurrent infections	93.94	6.06
Q26	Uncontrolled diabetes may cause psychological problems	89.74	10.26
Q27	Uncontrolled diabetes may cause erectile dysfunction/ low libido	93.01	6.99
Q28	A urine glucose test is helpful in diagnosis of type 2 diabetes	95.34	4.66
Q29	A blood sugar test is helpful in diagnosis of type 2 diabetes	96.74	3.26
Q30	HbA1c test is a better monitoring index of blood glucose fluctuations	96.27	3.73
Q31	Fasting blood sugar level above 125 mg/dL suggest diabetes	86.01	13.99
Q32	Random blood sugar level of 200 mg/dL or higher suggest diabetes	89.04	10.96
Q33	Balanced diet and exercise are the preferred treatment for type 2 diabetes	96.04	3.96
**Q34**	Oral hypoglycemic drugs are used for the treatment of type 2 diabetes	36.36	63.64
Q35	Insulin is used for the treatment of diabetes	93.01	6.99
Q36	Increased physical exercise decreases the chances of developing diabetes	96.04	3.96
Q37	Weight reduction in overweight individuals decreases the risk of developing diabetes	96.74	3.26
Q38	Quitting tobacco decreases the chances of developing diabetes	84.38	15.62
Q39	Quitting alcohol decreases the chances of developing diabetes	91.38	8.62
Q40	Increased intake of vegetable decreases the chances of developing diabetes	91.14	8.86
Q41	Reducing intake of sugary food decreases the chances of developing diabetes	92.31	7.69
Q42	Reduced carbohydrate intake decreases the chances of developing diabetes	91.61	8.39
Q43	Eating low fat food decreases the chances of developing diabetes	86.01	13.99
Q44	Reduced total calorie intake decreases the chances of developing diabetes	86.71	13.29
**Q45**	If one is going to be diabetic (prediabetic), there is not much he can do about it	13.29	86.71
Q46	People who make a good effort to control the risk of developing diabetes are less likely to develop diabetes	92.31	7.69
Q47	If people don't change their lifestyle, such as diet and exercise, they are at a risk of developing diabetes over the next 10 years	93.94	6.06

Note: Questions with a lower percentage of “Correct” answers are highlighted in bold.

Most participants recognized that diabetes is preventable (96.27%) but not curable (76.79%). However, a large majority (86.71%) responded negatively (“No”) to the notion that prediabetes is reversible or that no preventive measures can be taken for individuals with prediabetes.

Participants correctly identified common symptoms of diabetes, including frequent urination (97.2%), slow wound healing (95.8%), and excessive thirst (91.1%). Conversely, only 27.74% of participants were aware that excessive eating is also frequently linked to diabetes.

Participants demonstrate strong awareness of key risk factors for diabetes, including obesity (97.44%), family history (94.87%), and age (90.91%). However, knowledge about ethnicity as a contributing risk factor was notably lower, with only 29.37% recognizing its relevance. Understanding of insulin as a treatment for diabetes was high (93.01%), yet a significant portion of participants (63.64%) was unfamiliar with the role of oral hypoglycemic medications in diabetes management.

Several variables were found to be associated with participant's responses to the diabetes knowledge questions. For example, the determination of whether diabetes is characterized by high blood glucose (Q1) was influenced by students' sources of information and their eating habits. Those who had received education through targeted programs and maintained healthy eating pattern were more likely to answer Q1 correctly.

Most students responded negatively to the question regarding excessive eating occurs in patients with diabetes (Q3). Students who obtained information from books and magazines were significantly more likely to respond affirmatively to the question than those who did not consult these sources. Age also played a role. Students over 20 years old were more likely to believe that diabetes could be treated compared with younger students. .

For Q4, both gender and source of information showed negative correlations. Students whose primary source of information about type 2 diabetes was social education programs were more likely to answer this question incorrectly. Similarly, male students were more likely than females to respond incorrectly. Comparable patterns were observed across many of the remaining 45 diabetes knowledge questions.

Table 3 contains the results from the logistic regressions applied to each of the 47 questions, the regression coefficients, their associated *p* -values, the odds ratio (OR), and the relevant 95% confidence interval for the OR.

**Table 3:** Demographic questions corresponding to each item assessing diabetes-related knowledge

Question	Demographic variables statistically associated to the question	Estimate	*p* -Value	OR	95% CI for OR	
Q1	My main source of information about type 2 diabetes is from social awareness programs	0.556	0.012	1.743	1.132	2.684
	Are you maintaining healthy diet?	0.616	0.034	1.852	1.049	3.268
Q2	Height	0.09	0.006	1.094	1.026	1.166
	Does any of your family member or relative has/had diabetes mellitus?	1.449	0.007	4.26	1.495	12.14
Q3	Age:	0.992	0.007	2.697	1.304	5.577
	My main source of information about type 2 diabetes are newspaper, books, and magazines	0.598	0.016	1.819	1.115	2.967
Q4	My main source of information about type 2 diabetes is from social awareness programs	–0.773	0.028	0.461	0.231	0.922
	Gender: Male	–0.83	0.045	0.436	0.194	0.98
Q5	My main source of information about type 2 diabetes is from the awareness given in the schools	–0.789	0.004	0.454	0.266	0.775
	Age	1.288	0.084	3.627	0.841	15.636
	My main source of information about type 2 diabetes are scientific lectures and seminars	0.565	0.054	1.759	0.99	3.123
Q6	Gender: Male	–0.811	0	0.444	0.284	0.695
	Do you suffer from diabetes mellitus?	2.439	0.027	11.462	1.328	98.947
	Year of study	0.499	0.028	1.646	1.056	2.568
	My main source of information about type 2 diabetes is from social awareness programs	0.482	0.033	1.619	1.039	2.521
Q7 a						
Q8	My main source of information about type 2 diabetes is from social awareness programs	–1.296	0.054	0.274	0.073	1.025
Q9	Gender: Male	–1.877	0.002	0.153	0.046	0.509
Q10 a						
Q11 a						
Q12	My main source of information about type 2 diabetes is social media	0.777	0.013	2.175	1.177	4.018
	Gender: Male	0.623	0.045	1.865	1.015	3.429
Q13	Age	–1.67	0	0.188	0.078	0.455
	Gender: Male	0.998	0.007	2.713	1.308	5.625
Q14	You are currently a:	–0.56	0.04	0.571	0.335	0.974
Q15	Age	–2.021	0.003	0.132	0.035	0.508
	Do you suffer from diabetes mellitus?	–2.946	0.002	0.053	0.008	0.349
Q16 a						
Q17 a						
Q18	Age	–1.079	0.02	0.34	0.137	0.842
Q19	My main source of information about type 2 diabetes are scientific lectures and seminars	0.551	0.011	1.735	1.136	2.65
Q20	Does any of your family member or relative has/had diabetes mellitus?	0.916	0.02	2.5	1.157	5.402
Q21	Gender: Male	0.736	0.035	2.088	1.051	4.149
Q22	My main source of information about type 2 diabetes are my friends and relatives	1.624	0	5.073	2.653	9.699
	My main source of information about type 2 diabetes is from the awareness given in the schools	–1.044	0.002	0.352	0.184	0.674
	You are currently a:	–0.674	0.038	0.51	0.27	0.963
Q23	You are currently a:	–0.712	0.025	0.491	0.263	0.915
Q24	My main source of information about type 2 diabetes are scientific lectures and seminars	–0.826	0.026	0.438	0.211	0.907
Q25	Weight	0.029	0.032	1.03	1.003	1.058
	My main source of information about type 2 diabetes is from social awareness programs	–0.85	0.046	0.428	0.185	0.987
Q26 a						
Q27	Does any of your family member or relative has/had diabetes mellitus?	–1.92	0.061	0.147	0.02	1.093
Q28	Weight	0.027	0.074	1.028	0.997	1.059
Q29	Does any of your family member or relative has/had diabetes mellitus?	1.355	0.016	3.876	1.287	11.674
	Height	0.066	0.045	1.068	1.002	1.139
Q30	My main source of information about type 2 diabetes are scientific lectures and seminars	–1.062	0.044	0.346	0.123	0.97
Q31 a						
Q32	Does any of your family member or relative has/had diabetes mellitus?	–1.26	0.039	0.284	0.086	0.939
Q33 a						
Q34	Gender: Male	–1.212	0	0.298	0.186	0.477
	My main source of information about type 2 diabetes are scientific lectures and seminars	0.661	0.002	1.937	1.274	2.946
	Weight	0.013	0.023	1.013	1.002	1.024
Q35	Are you engaged in any physical exercises (150 min/week)?	1.083	0.031	2.953	1.103	7.905
	Gender: Male	–0.906	0.054	0.404	0.161	1.016
Q36 a						
Q37	Weight	0.055	0.016	1.057	1.01	1.105
Q38	My main source of information about type 2 diabetes are scientific lectures and seminars	–0.573	0.032	0.564	0.333	0.953
Q39	You are currently a:	–0.712	0.042	0.491	0.247	0.974
	My main source of information about type 2 diabetes is social media	0.726	0.039	2.067	1.039	4.11
Q40	Weight	–0.018	0.015	0.983	0.969	0.997
Q41 a						
Q42	Age	16.266	0.988	Inf	0	Inf
Q43	Age	1.681	0.102	5.373	0.718	40.196
	My main source of information about type 2 diabetes are newspaper, books and magazines	0.729	0.056	2.072	0.981	4.375
Q44	Age	1.696	0.098	5.451	0.731	40.661
Q45	My main source of information about type 2 diabetes are newspaper, books and magazines	1.137	0	3.119	1.709	5.691
	My main source of information about type 2 diabetes is from social awareness programs	1.101	0.001	3.006	1.614	5.599
	Are you engaged in any physical exercises?	–1.01	0.007	0.364	0.174	0.763
	Are you maintaining healthy eating?	0.89	0.015	2.434	1.186	4.996
Q46	Weight	0.031	0.012	1.032	1.007	1.057
Q47 a						

Abbreviations: CI, confidence interval; OR, odds ratio.

a No demographic variable was found to be statistically significantly associated with it.

**Fig. 1:**
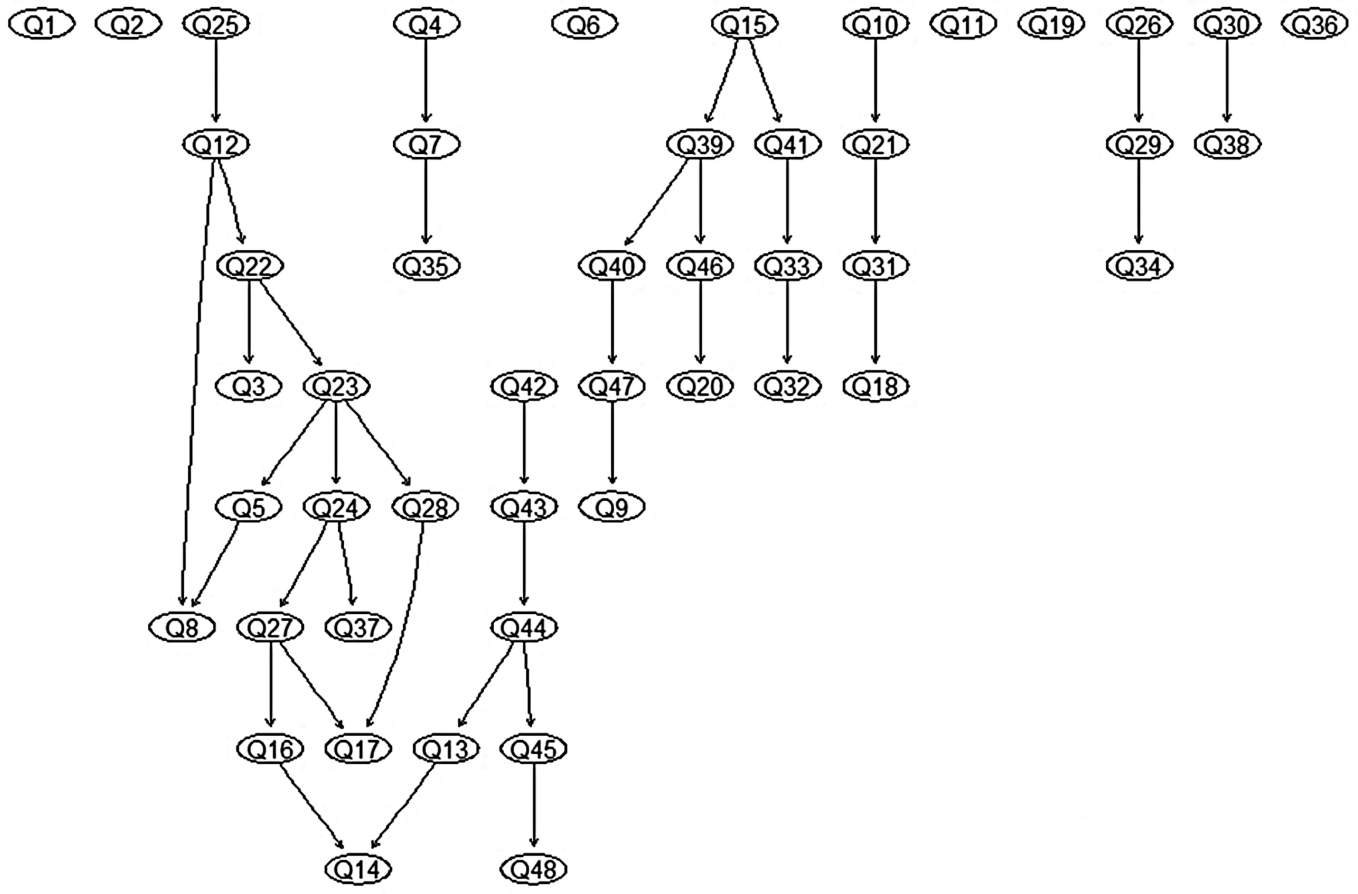
Bayesian network of the 48 questions of interest.

Several knowledge questions were found to be interrelated. Fig. 1 shows the network analysis of these questions, where each question is represented by an ellipse within the diagram. For example, questions 7 and 24 are linked to question 11, which in turn is connected to questions 21. In contrast, questions 1, 5, 10, 18, and 35 are not connected to any other questions and thus appear isolated within the network.

The arrows indicate the direction of the relationships; however, these should be interpreted with caution, as the analysis is exploratory and not based on expert knowledge. At this stage, it is more meaningful to focus on the presence of relationships between questions rather than the direction of those relationships.

## Discussion

To address the global type 2 diabetes epidemic, 14 this study assessed preprofessional health science students' (premedical, predental, prepharmacy, and preapplied medical science) knowledge of the disease. A total of 429 students participated, where majority of whom were aged 20 years or younger. Overall, the findings revealed a satisfactory level of general knowledge about type 2 diabetes. .

Questions related to diabetes prevention and the potential for a cure received the highest percentage of accurate responses, with prevention scoring slightly higher. Although most students responded accurately, a few minor gaps in knowledge were identified. Specifically, students exhibited limited awareness regarding the association between diabetes and eating disorders, the influence of ethnicity on diabetes prevalence, and the role of oral hypoglycemic agents in diabetes management. Students' sources of information, as well as their dietary habits and physical activity, appeared to influence the quality and accuracy of their diabetes knowledge.

It is commonly assumed that students of health sciences possess substantial knowledge about health-related matters, leading others to seek their advice, even though these students are still in training.

The questions on diabetes prevention received the highest proportion of affirmative responses, closely followed by those concerning the potential for curing diabetes. The results of this study align with previous findings. A study conducted in the United Arab Emirates reported that college students had a good knowledge of diabetes prevention, 5 while a study in China similarly showed high correct responses rates on prevention-related questions. 6

The prevalence of diabetes among the adolescent population in Saudi Arabia has increased significantly over the past two decades. 15 One way to reduce this prevalence is through education about risk factors and prevention strategies. Over the past decade, various public health campaigns have been launched in Saudi Arabia to counter the diabetes epidemic. 15 16 Although this study did not directly evaluate the impact of these initiatives, it is possible that they contributed to students' awareness of diabetes prevention.

In this study, deficiencies in knowledge about type 2 diabetes were identified in the study population. First, students did not recognize ethnicity as a significant factor influencing diabetes occurrence. Lifestyle and environmental factors are likely the main drivers of this trend. However, published literature indicates that diabetes risk varies by ethnicity. 17 Arab populations, particularly in Middle Eastern countries, show some of the highest projected increases in diabetes worldwide, with prevalence expected to rise by more than 90%. 18 It is therefore important to incorporate information on the role of ethnicity into diabetes educational programs in Saudi Arabia, both to improve students understanding and to reduce the risk of diabetes in the population.

Students also reported a lack of knowledge about diabetes treatment. While most were aware of insulin therapy, they showed insufficient understanding of oral antidiabetic medications. This may arise from difficulty differentiating between type 1 and type 2 diabetes and their respective treatment strategies. This finding is concerning given that students had received introductory lectures on diabetes as part of their preprofessional education. Our findings suggest that the material was not covered in sufficient depth, or students did not engage adequately with the content. Both possibilities highlight the need for curricular reinforcement to ensure students acquire a solid understanding of diabetes management. One of the most important findings of this study is that the source of information significantly influences both the level of knowledge and the accuracy of information regarding diabetes. For example, students who relied primarily on social education programs were more likely to give incorrect answers. While social education programs can be effective if based on sound evidence, many campaigns particularly those shared online contain unverified or outdated content. Such programs may fail to achieve their objectives and, in some cases, may disseminate misinformation.

To address this, there is a clear need for expert-driven, evidences-based health campaigns. In Saudi Arabia, health care professionals are highly trusted source of medical advice, 19 and their involvement in educational initiatives could improve campaign effectiveness and credibility. 20

## Limitations

The findings of this study should be interpreted in light of some limitations. First, the sample was restricted to students from a single college, which may limit the generalizability of results. Second, the relatively large number of knowledge questions compared to other studies, 5 6 7 which may have increased the students' cognitive load. Third, because the questionnaire was self-administered, it is unclear whether all participants fully comprehended or correctly interpreted the questions. Finally, the correlations between knowledge questions identified through Bayesian network analysis should be considered exploratory and do not imply a causal relationship.

## Conclusion

This study shows that health science students generally possess a satisfactory level of knowledge regarding type 2 diabetes, particularly in relation to its prevention. However, notable gaps remain, especially regarding the role of ethnicity in diabetes risk, the use of oral medications in diabetes treatment, and the association between diabetes and eating disorder. These findings point to areas where curricular enhancement is needed to ensure that students are adequately informed about the nature of diabetes management. The study also highlights the influence of information sources on student knowledge, with those relying on unregulated social educational programs more likely to provide inaccurate responses, emphasizing the importance of expert-led, evidence-based public health initiatives, especially in the era of social media. .

Given the rising prevalence of diabetes in Saudi Arabia, and the high level of public trust in health care professionals, it is crucial to equip future health care professionals with comprehensive and accurate knowledge of the disease. While this study offers valuable insights, the limitations noted should be kept in mind when interpreting the results.
